# Low Rates of Immunity among Medical Students and Residents in the Era of the Resurgence of Measles

**DOI:** 10.3390/pathogens13090784

**Published:** 2024-09-11

**Authors:** Cristiana Ferrari, Giuseppina Somma, Vittorio Caputi, Michele Treglia, Margherita Pallocci, Fabian Cenko, Ersilia Buonomo, Mariachiara Carestia, Luca Di Giampaolo, Ole F. Olesen, Luca Coppeta

**Affiliations:** 1 PhD Program in Social, Occupational and Medico-Legal Sciences, Department of Occupational, Medicine, University of Rome Tor Vergata, Viale Oxford 81, 00133 Rome, Italy; 2 Department of Biomedicine and Prevention, University of Rome Tor Vergata, Viale Oxford 81, 00133 Rome, Italy; giuseppina.somma@ptvonline.it (G.S.); vittorio.caputi@gmail.com (V.C.); michelemario@hotmail.it (M.T.); margherita.pallocci@gmail.com (M.P.); lcoppeta@gmail.com (L.C.); 3 Faculty of Medicine, Catholic University of “Our Lady of Good Counsel”, 1000 Tirana, Albania; f.cenko@unizkm.al (F.C.); ersiliabuonomo@gmail.com (E.B.); mariachiara.carestia@uniroma2.it (M.C.); 4 Department of Occupational Medicine, University of Chieti “G. D’Annunzio”, 66100 Chieti, Italy; l.digiampaolo@unich.it; 5 European Vaccine Initiative, Universitätsklinikum Heidelberg (Heidelberg University Hospital), Voßstraße 2, 69115 Heidelberg, Germany; ole.olesen@euvaccine.eu

**Keywords:** measles, medical students, healthcare workers, measles outbreaks, immunity, vaccine

## Abstract

Measles is a highly contagious viral disease spread through respiratory droplets. The number of reported cases increased worldwide in 2023, particularly in the European Region. Italy reported 213 cases in the first quarter of 2024, with most of them in unvaccinated adults aged 15–64. Maintaining high vaccination coverage is essential to prevent outbreaks, especially in healthcare settings where measles transmission is a significant risk. In our study, we collected serological and demographic information from all Italian and foreign medical students and residents (850) who underwent a pre-training assessment at the Tor Vergata Occupational Medicine Service, Rome, between 3 April 2023 and 31 January 2024. Of the 850 students and residents analyzed, we found only 546 (64.2%) with a protective level of IgG antibodies against measles, with a median IgG level of 2.00 AI. A significant proportion of students and residents were serologically non-immune, raising concerns about the potential risk of hospital transmission. To manage this risk, it is important to assess serological levels, vaccinate those with inadequate levels, and promote vaccination in the general population.

## 1. Introduction

Measles is a highly contagious viral disease caused by a single-strand RNA virus of the genus Morbillivirus and the family Paramyxoviridae. It is an acute viral disease that spreads easily from person to person through respiratory droplets. As the disease progresses, distinctive white lesions known as Koplik’s spots may appear on the buccal mucosa before a rash develops. Unfortunately, due to a temporary weakening of the immune system, measles can lead to bacterial superinfections, resulting in complications such as pneumonia, otitis, bronchitis, and diarrhea. More serious complications, including encephalitis and subacute sclerosing panencephalitis (SSPE), can also affect the nervous system [1].

In 2023, there was a significant increase in reported measles cases and outbreaks worldwide compared with previous years. In the EU/EEA (European Union and European Economic Area) alone, 2361 cases were reported, of which the majority (1607, 68%) were laboratory-confirmed. This was in stark contrast to the 123 and 61 cases reported in the same area in 2022 and 2021, respectively. The overall reporting rate in 2023 was 5.2 cases per 1,000,000 population, a significant increase from the rates observed in 2020–2022 (0.3 in 2022, 0.1 in 2021 and 4.3 in 2020), but still lower than the rate observed in 2019 prior to the SARS-CoV2 pandemic (27.2) [2].

The wider European Region experienced a similar surge in measles cases, with over 30,000 reported by 40 out of the 53 countries, leading to approximately 21,000 hospitalizations [3]. Experts predict that these numbers are only expected to increase in 2024 due to declining vaccination coverage seen during the pandemic period [4].

The recent increase in measles cases is of great concern because of the potential public health impact. It is also a reminder of the critical importance of maintaining high vaccination coverage to prevent outbreaks of measles. In this context, promoting vaccination and raising awareness of the serious consequences of measles are essential components in the fight against this potentially deadly disease.

In January and February 2024, the European Centre for Disease Prevention and Control (ECDC) reported an increasing number of measles cases in EU/EEA countries, with six fatal cases reported in Romania and one in Ireland [5].

In the first quarter of 2024, Italy reported 213 measles cases, corresponding to a national incidence of 14.5 cases per million inhabitants. Fifteen out of twenty Italian regions reported measles cases, but 68% of cases were reported in Lazio, Sicily, and Tuscany, with the highest incidence in Lazio (44.9/million). The average age of the cases was 31 years, and three quarters of cases (74.2%) were aged between 15 and 64 years. Of the 213 measles cases, 181 were laboratory confirmed, 9 were probable, and 23 were possible. Eighteen of the reported cases (8.4%) were imported cases. Vaccination status is known for 187 of the 213 cases (87.8%). Of these, 165 (88.2%) had not been vaccinated prior to infection. The remaining 11 cases received a single dose of vaccine, while nine individuals were vaccinated with two doses. In two cases, the number of doses administered is unknown [6].

Given the high basic reproductive number (R0) of measles (12–18), achieving and sustaining the elimination of endemic measles requires the achievement of extremely high levels of community immunity (>95% vaccination coverage) and the implementation of effective laboratory-based surveillance to detect and control outbreaks promptly [7]. In a published study, Lo and Hotez showed a 5% decrease in vaccine coverage results in a threefold increase in measles cases [8].

Even before the pandemic, only a small number of countries had achieved at least 95% vaccination coverage with both the first and second doses of measles-containing vaccine. Furthermore, the number of countries meeting these targets declined further during the pandemic. These low coverage estimates indicate that routine childhood immunization against measles in most countries is below the recommended levels needed to achieve and sustain measles elimination [9].

Several studies have shown that healthcare workers (HCWs) face a higher risk of exposure to the measles virus than non-HCWs [2,3]. HCWs thus accounted for 33.3% of measles cases in Italy in 2022 [10]. In recent years, there has been an increasing trend for measles outbreaks occurring in healthcare settings, accounting for up to 50% of reported cases [11]. Measles transmission in healthcare settings poses a particular threat to vulnerable groups, including infants and immunocompromised individuals. To prevent nosocomial outbreaks, it is crucial to ensure adequate vaccination coverage and high levels of immunity among healthcare workers (HCWs). The World Health Organization (WHO) recommends that all HCWs in direct contact with patients should be immune to measles through vaccination or previous infection [12,13].

The aim of this study was to evaluate the susceptibility of medical students and residents of a teaching hospital in Rome to measles infection. This was carried out by performing serological assessments of their immune status.

## 2. Materials and Methods

To determine the serological status of medical students and residents starting their internship at the teaching Hospital Tor Vergata in Rome, we collected serological and demographic information from all subjects who underwent a pre-training assessment at the Occupational Medicine Service between 3 April 2023 and 31 January 2024. All participants underwent venipuncture to obtain a 10 mL blood sample from either the cephalic vein, median cubital vein, or basilic vein for routine blood testing. Blood samples were analyzed for the presence of measles-specific IgG antibodies using the Alifax VIR-CLIA^®^ KIT VIR VCM054 Measles IgG assay. This assay uses chemiluminescent immunoassay (CLIA) technology, offering high sensitivity and specificity values. Serum IgG levels were expressed as antibodies index (AI) = sample RLU (Relative luminescence)/calibrator RLU. According to the manufacturers’ instructions, the values of the antibodies index were considered negative if <0.9, equivocal if 0.9–1.1, and a value >1.1 was considered positive. Results were extracted from Modulab^®^ laboratory software (3.1.02 (build 4)). Personal information, including name, age, sex, and nationality, was collected for each participant. Vaccination data were not collected due to limited availability, as not all subjects were able to provide their personal vaccination schedule and Italy does not have a central vaccination registry.

All of the subjects underwent a physical examination and their medical history was taken. Participants with incomplete serological data or positive results for measles-specific IgM antibodies were excluded. Median IgG antibody levels in protected students and residents were compared by age group using multivariate analysis.

Statistical analysis was performed to describe the characteristics of the subjects in terms of numbers and percentages for different types of variables. Differences in percentages between groups were assessed using the chi-squared test. Two-tailed *t*-tests were used to compare continuous variables, while Pearson’s correlation tests were used to assess the statistical relationship between continuous variables.

Statistical significance was considered at *p* values < 0.05. Statistical analyses were performed using the SPSS software version 25.0 for Windows. Prior to the start of the study, approval was obtained from the Ethics Committee for Research on Human Subjects of the Teaching Hospital of Rome Tor Vergata (approval no. 133/21).

## 3. Results

**Figure 1:**
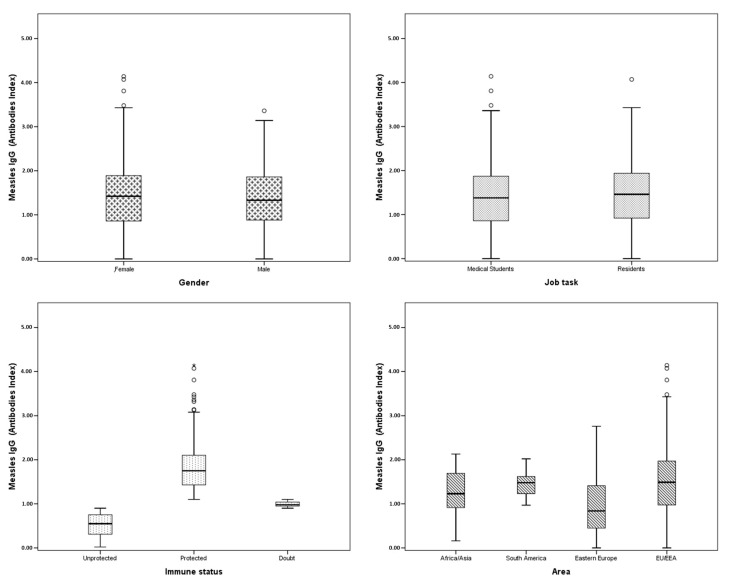
Measles IgG antibody index according to each characteristic (gender, job task, immune status, and area).

A total of 850 medical students and residents (267 males and 583 females) were assessed from their medical records. The mean age of the study group was 24.7 years (range: 18–30); the mean age of the females was 24.6 years; and that of the males was 24.9 years. Further, 669 (78.7%) were medical students, while 181 (21.3%) were residents. Of the total number of subjects, 687 (81.4%) were from the EU/EEA, 133 (15.8%) from Eastern Europe, 19 (2.2%) from Asia or Africa, and 5 (0.6%) from South America. The main characteristics of the population are shown in Table 1 and Figure 1.

A total of 546 medical students and residents (64.2%) showed protective IgG antibodies against measles, with a mean IgG level of 1.39 AI ± 0.71 AI. Female medical students and residents were more likely to be serologically immune than their male counterparts (65.7% vs. 61.0%, respectively), although this difference was not statistically significant (*p* = n.s.). The percentage of residents found to be protected was higher than that of the medical students, but this difference was not statistically significant (*p* = n.s.). Regarding the nationality of the subjects, medical students and residents from EU/EEA and South America were significantly more serologically immune than counterparts from other global area (*p* < 0.01, see Table 2). It is noteworthy that only ten subjects in total from Asia or Africa and South America were included in the study. The main results of the study are presented in Table 2.

## 4. Discussion

The primary objective of this study was to determine the percentage of individuals with serological immunity in a large group of medical students and residents doing their internship at the Tor Vergata Teaching Hospital in Rome. Our findings show that more than a third of the individuals in our study population lacked protective IgG levels against measles, highlighting the need for a comprehensive immunization program for young healthcare trainees. In contrast to the findings of previous studies [13,14,15], the present investigation showed no significant difference in measles antibody levels between males and females. The two groups exhibited comparable levels of IgG antibodies to measles. The consistency of the elevated incidence rates in young males indicates that the observed sex differences are more likely attributable to physiological and biological differences rather than behavioral factors. At older ages, differential exposure may be a contributing factor. These findings may provide further insights into the mechanisms of infection and inform the development of tailored vaccination schedules [16].

Subjects born after 2000 represent the population vaccinated by the compulsory vaccination program (Italian Legislative Decree 73 of 2017). It can therefore be reasonably assumed that Italian individuals born after 2000 represent a highly protected group [17]. Nevertheless, approximately 30% of the Italian study subjects in the age group of interest, for which vaccination is legally required, do not have serologic immunity.

Vaccination coverage in Italy against measles, mumps, and rubella was approximately 93.8% of children in 2021. These coverage levels were below those recommended by the WHO, which suggests an optimal level of 95% vaccine coverage to achieve herd immunity (also known as “population immunity”) [18].

The date and status of vaccination was not documented in the current study because not all subjects were able to provide their personal vaccination schedule and there is no central national vaccination registry in Italy. Consequently, it was not possible to calculate the time elapsed since vaccination, although this factor may be relevant in assessing the possible decline in antibody levels during the time since vaccination. In elimination settings, measles vaccine-induced immunity wanes over time, but not after natural exposure [19]. It is important to note that immunity to measles after two doses of the MMR vaccine is not lifelong. The antibody concentration declines below the protective threshold of 200 mIU/mL after about fifteen years (14.3 years to be exact) [20]. However, since the age of the individuals is a reasonable surrogate for the time elapsed between vaccination and serologic assessment, we performed a comparative analysis of different age groups to assess the contribution of waning immunity. Despite the significant age difference between the two groups (medical students and residents), neither the proportion of residents who were protected nor the median antibody level showed a statistically significant difference from that of the medical students. There are a number of challenges to the analysis of these data that make a straightforward interpretation difficult. As mentioned above, in 2017, Italy introduced the mandatory vaccination of all individuals under 18 years of age for school enrollment [17]. Consequently, these younger cohorts can be considered fully vaccinated. Nevertheless, although the incidence of measles in Italy has been low in recent decades, it cannot be excluded that some older students were not vaccinated in childhood and may have been naturally immunized, especially considering that some measles outbreaks occurred in Italy between 2017 and 2019. [6]. The results indicate that a relatively low proportion of individuals in the target age group, who should be vaccinated by law, have been immunized, especially in an elimination context, given the epidemiological circumstances in Italy. Previous studies have shown suboptimal vaccination coverage for several vaccine-preventable diseases among healthcare workers and medical students in Europe, especially those working in high-risk settings [13,14,15].

Recent outbreaks of measles among young medical students have raised concerns about the importance of vaccination in preventing the spread of this highly contagious disease. It is thus essential to increase awareness and increase access to vaccines to control the spread of measles [21].

The efficacy of vaccines is supported by recent studies showing that the measles vaccine stimulates the immune system to fight the infection, reducing the likelihood that the virus will cause fatal symptoms and allowing for the infection to be controlled, leading to full recovery. In line with the above proposals, routine childhood vaccination has also been proposed to provide bystander immunity in the context of novel coronavirus control. The global spread of the novel coronavirus (COVID-19) has caused significant disruption to immunization programs worldwide. However, it is now well established that the measles vaccine is highly effective in preventing the disease itself. This is achieved by stimulating innate immune cells and inducing antibodies with cross-neutralizing activity against several pathogens, including SARS-CoV-2 [22,23,24,25].

The present study demonstrated that a large proportion of medical students and residents can be considered immunologically non-protected against measles. Given the ongoing circulation of the measles virus, it is recommended that HCWs undergo serologic testing and that consideration be given to vaccinating healthcare workers who are not vaccinated or have low antibody levels. This approach has previously been demonstrated to be highly cost-effective by empirical evidence [6,26,27].

Of note, the proportion of non-immune individuals in this study population was higher than in previous studies (35.8% vs. 33.3% in 2022–2023) [27]. It is noteworthy that when analyzed by nationality, Italian subjects had a protection rate of 70.7%, while foreign-born subjects had a protection rate of 40.8%. In a previous study carried out in 2022–2023, the rate was 62.6% [28]. These levels are far from those expected in a population when a 95% vaccination rate, required for herd immunity, is achieved [29].

Given the high likelihood of measles epidemics affecting healthcare systems and the dual role of HCWs as potential sources of infection for vulnerable patients and as healthcare providers, it is imperative to prioritize vaccination of this population.

It is also recommended that occupational medicine services promote interventions to address vaccine hesitancy among young operators. A recent systematic review found high rates of low vaccination coverage and high rates of hesitancy for MMR among healthcare operators and medical students in Italy and in the rest of Europe [30]. Vaccine hesitancy is only one factor contributing to the decline in MMR vaccination rates. Navigating the healthcare system can be challenging, and it is incumbent upon us to improve accessibility. It is suggested that convenient appointments may increase uptake [30]. Workplace vaccination has been shown to be the most cost-effective approach to improving vaccination rates among HCWs and students and should be offered by occupational health services following pre-employment screening [26,31,32,33].

In addition, the Advisory Committee on Immunization Practices (ACIP) in Italy has recommended that in the event of an outbreak in a hospital’s community or within the hospital itself, all personnel with direct patient contact who were born in 1957 or later and who cannot provide documentation or proof of receipt of two doses of measles vaccine on or after their first birthday should be vaccinated against measles [34].

A possible limitation of this study is the lack of consideration of the different risks of exposure according to the different hospital settings. The likelihood of being infected with mumps varies in different areas of the hospital. Certain departments, such as emergency, infectious diseases, pediatrics, intensive care, oncology, and hematology, have a higher risk of exposure due to the specific patient populations they serve. However, given the relatively short tenure of these young trainees and residences, occupational exposure is unlikely. Another major limitation of the study was that, due to the retrospective nature of the survey and the lack of a freely available national vaccine registry, data on previous vaccination status were not available. We have partially addressed this limitation by considering the subjects’ age as the most reasonable proxy for the time of MMR vaccination. This approximation can be considered valid for Italian individuals subject to mandatory vaccination (born after 2000), while it may not be accurate for older subjects, as childhood vaccination coverage rates increased slowly from 1990 to 2000, always remaining below the 80% rate. Another limitation of this study is that no data were collected to examine differences in immunity conferred by different types of vaccines. It is plausible that there may be differences in immune response and efficacy depending on the type of vaccine administered. Furthermore, the inability to investigate immune memory cells, given that vaccines are predominantly administered during childhood, suggests the possibility of additional immune responses in the form of memory cells. Further study is required to analyze memory T/B cells.

## 5. Conclusions

The findings of this study indicate that measles immunity among Italian and foreign medical students and residents is still below optimal levels. It is imperative that serological testing and/or targeted vaccination strategies be implemented to ensure that all medical students and residents are adequately protected against measles before beginning their internships in hospital settings. It is clear that this younger generation represents a key target group for behavioral change interventions to improve vaccine uptake and coverage. It is recommended that occupational health services educate these young healthcare workers about the efficacy and safety of vaccines to achieve the highest possible vaccination rates. This will help to achieve the goal of 95% of the population being vaccinated, resulting in herd immunity. Since a low immunity rate among medical students could also reflect a low rate of immunization in the general population, it is crucial to evaluate possible public health strategies to respond a potential pandemic, such as active case surveillance, early isolation of active measles cases, exclusion from work and quarantining of contact individuals without evidence of immunity, and vaccination offer for all susceptible individuals.

## Figures and Tables

**Table 1:** Main characteristics (gender, tasks, IgG level, area) of the study population.

Characteristics	N (%)	Mean Age ± SD	Mean IgG Level ± SD
Gender			
Male	267 (31.4%)	24.9 ± 3.07	1.36 ± 0.68
Female	583 (68.6%)	24.6 ± 2.89	1.41 ± 0.72
Tasks			
Medical students	669 (78.7%)	23.6 ± 2.17	1.38 ± 0.70
Residents	181 (21.3%)	28.9 ± 0.85	1.44 ± 0.74
Measles-specific IgG level			
>1.1 AI	546 (64.2%)	24.8 ± 3.06	1.81 ± 0.49
0.9–1.1 AI	82 (9.7%)	24.7 ± 2.75	1.00 ± 0.06
<0.9 AI	222 (26.1%)	24.6 ± 2.75	0.51 ± 0.26
Area			
EU/EEA	687 (81.4%)	24.9 ± 3.10	1.48 ± 0.71
Eastern Europe	133 (15.8%)	24.1 ± 1.78	0.94 ± 0.64
Asia/Africa	19 (2.2%)	22.1 ± 2.30	1.24 ± 0.54
South America	5 (0.6%)	23.4 ± 2.07	1.49 ± 0.52

**Table 2:** Main findings (protection percentage, mean age, mean IgG level) according to gender, tasks, and area of the protected population.

Characteristics	N Protected (%)	Mean Age ± SD	Mean IgG Level
Gender			
Male	163 (61.0%)	24.7 ± 3.20	1.80 ± 0.45
Female	383 (65.7%)	24.8 ± 3.00	1.82 ± 0.50
Tasks			
Medical students	422 (63.1%)	23.5 ± 2.29	1.81 ± 0.48
Residents	124 (68.5%)	28.9 ± 0.85	1.82 ± 0.52
Area			
EU/EEA	484 (70.5%)	24.8 ± 3.12	1.83 ± 0.50
Eastern Europe	46 (34.6%)	25.0 ± 2.25	1.69 ± 0.41
Asia/Africa	12 (63.2%)	22.0 ± 2.41	1.53 ± 0.31
South America	4 (80.0%)	23.5 ± 2.38	1.58 ± 0.33

## Data Availability

Data available on request due to privacy restrictions.

## Statement

The present study was conducted as part of the European Alliance for Collaborative Research on Vaccine Confidence in Public and Occupational Health (EuCoRVaC).
